# The Use of Extended Reality Technologies in Sport Perceptual-Cognitive Skill Research: A Systematic Scoping Review

**DOI:** 10.1186/s40798-024-00794-6

**Published:** 2024-11-29

**Authors:** Aden Kittel, Riki Lindsay, Peter Le Noury, Luke Wilkins

**Affiliations:** 1 Centre for Sport Research, Institute for Physical Activity and Nutrition, Deakin University, Geelong, Australia; 2 School of Exercise and Nutrition Sciences, Deakin University, Geelong, Australia; 3 Institute of Education, Arts and Community, Federation University, Ballarat, Australia; 4 Australian Football League, Melbourne, Australia; 5 Sports, Performance, and Nutrition (SPAN) Research Group, La Trobe University, Bundoora, VIC Australia

**Keywords:** Virtual reality, Perceptual-cognitive, Decision-making, Extended reality, Sport

## Abstract

**Background:**

Extended Reality (XR) technologies, such as Virtual Reality (VR) and 360°VR are growing rapidly in the scientific literature and sporting practice. These have been used for a range of skills, particularly perceptual-cognitive skills. However, to our knowledge, there is no systematic scoping review on this topic identifying the current state of play of the research area by characteristics such as study type, technology type, or sport investigated, and such a review would help guide the future direction of this area. Therefore, this study aimed to systematically review the extent of XR technology in sport for assessing and training athletes’ and officials’ perceptual-cognitive skills.

**Methods:**

Electronic databases (SCOPUS, Web of Science, SPORTDiscus, PsycINFO) were searched for relevant articles up until January 2024. Studies were included if they used XR technologies to assess or develop sport-specific, higher order perceptual-cognitive skills.

**Results:**

57 studies met the inclusion criteria for this review, of which 67% were published from 2020. Most studies conducted quantitative research designs, with 66% of studies adopting a cross-sectional assessment approach and 28% conducting an intervention to assess performance improvements. Decision-making was the most prevalent skill investigated, across 60% of studies. The most common technology was head mounted display (51%) presenting animated environments and the most common sports investigated were football and handball (32% and 19% of studies, respectively).

**Conclusions:**

This review highlights a significant growth in the research exploring XR technologies in sport for perceptual-cognitive skill development and understanding, with most studies published in the last 4 years. Prominent technology types (e.g. animated HMD), perceptual-cognitive skills (e.g. decision making), study designs (e.g. quantitative assessment), and sports (e.g. football) are identified and discussed along with practical implications and future research.

**Key Points:**

- Extended reality technologies for sports perceptual-cognitive skills is an emerging field, marked by key trends in the types of technology used and the perceptual-cognitive skills being studied.
- Decision-making is the most commonly studied perceptual-cognitive skill, and these technologies report to have high representativeness and engagement when being used.
- More research is required to explore the effectiveness of this technology through intervention study designs, and further understand how it can be used and the perceptual-cognitive processes through qualitative research designs.

## Background

Adopting extended reality (XR) technology for training athletes and officials' expertise in sport presents an exciting development that has seen rapid growth over the past decade. XR refers to the umbrella term encompassing different types of computer-generated simulations, including virtual reality (VR), augmented reality (AR), 360°VR (also termed immersive video, 360° video [1]) and mixed reality (MR) [2]. The rapid growth and adoption of XR in sport can be attributed to multiple factors, including software and hardware advancements, and increased collaboration between technology companies and sports entities [3]. Another significant contributing factor has been compelling evidence from other domains, including the military, medicine and psychology, whereby XR training has produced positive effects on performance [4]. XR's appeal also lies in its capacity to manipulate constraints in complex and dynamic environments, facilitating the creation of specific, repeatable situations. Research has found that athletes report high perceived usefulness of XR, particularly for dynamic sports that require anticipation and decisions, such as rugby and basketball [5].

XR technology has been successfully applied in areas such as medicine, military operations, workforce training, emergency response, and education [4]. However, despite robust research evidence supporting the use of XR technology in these domains, its application in sports, particularly for training the perceptual-cognitive skill of athletes and officials, has been lacking [2]. Although XR technology has begun to make inroads into high performance sport, its adoption has outpaced scientific research, leading to a gap between its practical application and empirical understanding of its potential to enhance perceptual-cognitive skills. Consequently, the possible benefits of employing XR training tools in sport risk being undermined if not first underpinned by vigorous research evidence [2, 6]. Therefore, further research is necessary to comprehensively assess the extent and depth of studies investigating XR technology in sports expertise. This will offer stakeholders a more informed perspective on the status of research in this field and will enable researchers to understand the areas within the field of XR technology and sport that warrant further investigation. Given perceptual-cognitive skills are a fundamental component of achieving excellence in sports performance (including officials as well as athletes) [7], it is crucial that key stakeholders gain a comprehensive understanding of the current state of the research evidence around the use of XR training tools. This is pertinent given the complexity of decision-making in sport, and the possibilities XR technologies have for assessing and developing perceptual-cognitive skills [8].

It should be noted that several publications have reviewed the use of VR and XR technologies in sports training including systematic reviews of their application in decision-making training [9], athlete performance enhancement [10], and perceptual-cognitive skill development using video-based tools [11]. Additionally, Le Noury et al. [2] and Richlan et al. [12] provide comprehensive overviews of XR’s potential to revolutionize sports training, assessing both the benefits and limitations of these immersive technologies. Crucially, however, to our knowledge no review has yet provided an overview of research on XR for perceptual-cognitive skills in sports, specifically distinguishing between different perceptual-cognitive skills, study designs, types of XR technology, and the characteristics of the sports and participants involved. Indeed, presenting this information in this scoping review provides an efficient way for stakeholders in sport to gain further clarity on the current state of XR research in sport and helps them understand how they could utilise XR in the future. To ensure the effective integration of XR technologies in sport, it is essential to not only review the current state of research, but also the applied methodologies that can maximize skill transfer from virtual environments to real-world performance.

To address this, a principled approach, guided by ecological dynamics, provides a theoretical foundation for evaluating both the technology and the design of tasks. Ecological dynamics delves into the intricate relationship between individuals and their environment, emphasizing the significance of understanding whether acquired skills can seamlessly transition across varied settings, such as from XR simulations to actual competitive scenarios [13]. Translating these theoretical constructs into practical application, several applied frameworks, including representative learning design (RLD), affective learning design (ALD), and the modified perceptual training framework (MPTF), play a pivotal role [2]. RLD focuses on creating practice environments that faithfully replicate the perceptual and motor demands of real-world performance situations. ALD delves into the affective or emotional aspects of learning, exploring how XR interventions can influence athletes' emotional states and, consequently, their skill acquisition. Janssen et al. [8] provide a succinct framework for future design principles using VR technologies for representative decision-making, including ensuring the movements, interactions, viewpoint and scenarios are all representative. This naturally leads to a trade-off between XR technologies such as VR and 360°VR, where VR can be enhanced to allow participants to move and interact with the virtual environment as they could in a game, but the virtual video will not be real-world video such as 360°VR [1, 8]. The MPTF, on the other hand, refines the design of perceptual training tasks within XR environments, aligning them with principles derived from ecological dynamics [14, 15]. This holistic approach empowers professionals in the sports industry (e.g., coaches) to design XR-based training tools effectively and evaluate the potential for positive skill transfer to real-world performance settings. Furthermore, Broadbent et al. [16] highlight the importance of perceptual-cognitive skill training transferring to competition, which XR may provide an appropriate tool to achieve.

This review will focus on original research that has used XR as a tool for *training* perceptual-cognitive skills in athletes and officials, and original research that has assessed the reliability, validity, representativeness, and/or user experience of XR tools aimed at *understanding* perceptual-cognitive skill. In this review, we differentiate perceptual-cognitive skills from motor skills performed in a closed environment. As discussed by Bruce et al. [17], perceptual-cognitive relates to the skill of perceiving and understanding information from the environment, whereas perceptual-motor involves completing an action (i.e., doing) based on information. As such, the perceptual-cognitive skills within this paper are defined within the framework by Hodges et al. [18] as being high level and cognitive. These include attention/reaction time and eye movement (high level), and anticipation, decision-making, memory/knowledge, and executive functions (cognitive). For clarity for the reader, we have termed these perceptual-cognitive skills in the current review (i.e., decision-making, anticipation etc.), but they may have a motor component. However, the main focus is on the perceptual-cognitive element (i.e., understanding information from the environment).

The aim of this review is to investigate the scope of literature pertaining to the utility of XR technology in sport for assessing and training athletes and officials’ perceptual-cognitive skills. Given the importance of perceptual-cognitive skills in sport expertise and performance [12], and the growing rate of XR technology used to assess and develop these skills [2], it is important for practitioners and researchers to have a review that consolidates the existing work in this area. Importantly, this paper will provide essential information about each original research paper including the participants, sport being investigated, skill being investigated, type of XR technology that was used, study design information such as whether it was a qualitative, quantitative, mixed methods, intervention or validation study, and the conclusions identified. In doing so, this scoping review will provide key stakeholders with vital information to assist with their decision-making around how to utilise XR technology in their performance programmes. It will also highlight the potential areas in which XR technology could be utilised in the future to enhance athletes and officials’ performance, identifying trends within the literature regarding methodological approaches. Finally, this scoping review fills a gap in the research literature by comprehensively assessing the original research around the use of XR technology for athletes and officials, specifically aimed at perceptual-cognitive skills, which, to the best of our knowledge, has not yet been achieved.

## Methods

**Fig. 1:**
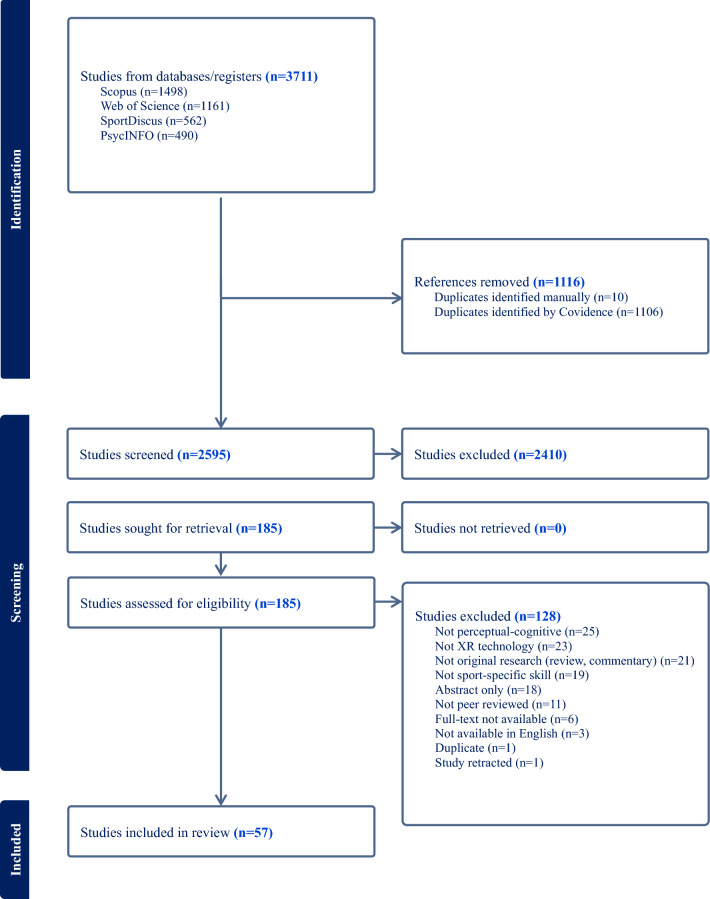
PRISMA flowchart of search process

The present review was conducted using a systematic approach according to PRISMA-P (Preferred Reporting Items for Systematic review and Meta-Analysis Protocols guidelines) [19], summarized in Fig. 1.

### Search Strategy

Electronic databases (SCOPUS, Web of Science, SPORTDiscus, PsycINFO) were initially searched on May 31, 2023, with an updated search conducted to incorporate additional articles up to January 2024. Search terms were (“Extended reality” OR “Virtual reality” OR “Immersive video” OR “360 video” OR “Augmented reality” OR “Mixed reality” OR “Virtual environment” OR “Simul*”) AND (“Perceptual-cognitive” OR “Perceptual-motor” OR “Decision-making” OR “Anticipation” OR “Pattern recognition” OR “Skill”) AND (Sport). Search terms were developed by the authors with experience publishing in this area.

### Inclusion and Exclusion of Studies

In line with the PICO approach [20] inclusion criteria were selected as follows:

I. Participants: Studies containing healthy individuals of any age undertaking any perceptual-cognitive skill within a sporting context were included. Perceptual-cognitive skills included were defined as being high level and above perceptual-cognitive skills [18].
II. Intervention: Studies were included if they investigated the use of XR technology on perceptual-cognitive skills in sport, including original research. The four types of original research categorised for this review are Quantitative (Assessment) (e.g., validity/reliability studies), Quantitative (Intervention) (e.g., training studies), Qualitative (e.g. interview studies), and Mixed-methods (i.e., Quantitative and Qualitative).
III. Comparison: No comparison necessary.
IV. Outcome: Studies need to include an outcome measure related to the effectiveness or validity of XR technologies related to perceptual-cognitive skills in sport.

In addition, non-peer reviewed literature, systematic reviews and literature reviews, and studies not originally written in English were excluded from the present review.

### Screening Strategy and Study Selection

Articles obtained from the electronic databases were stored using Covidence online systematic review software (Covidence, Veritas Health Innovation Ltd., Melbourne, Australia) for screening. Duplicates from the use of multiple databases were identified within Covidence and removed. The screening process was conducted by two authors (AK and RL) and was blinded. At the conclusion of the screening phase, any discrepancies were resolved by a third author (LW).

### Data Extraction

Following screening, two authors (AK and LW) extracted relevant data from the included full texts into a customised data extraction form in Covidence. Discrepancies in the data extraction form were discussed with a third author (RL) until consensus was reached. Perceptual-cognitive skills identified in this review were defined by Hodges et al. [18].

## Results

### Search Results

A total of 3711 articles were identified across four databases, with 2595 articles remaining after the removal of duplicates. Following the screening of 186 full-texts, 57 articles were included in the final review. The study search and selection process are presented in Fig. 1.

### Demographic Characteristics of Included Studies

Key results are summarised in Table 1. The total population was 1810. In the studies identified, sample sizes ranged from one to 127, with most studies examining sample sizes of 20–39 participants (*n* = 28). Regarding sex, the most prevalent sample was a mix of males and females (*n* = 29), followed by male only (*n* = 15) and female only (*n* = 1). Twelve studies did not report sex. Most participants were classified as athletes (*n* = 51), followed by officials (*n* = 6). In the studies investigating athlete populations, most studies reported mixed samples, comparing a range of skill levels (n = 23). Of the athlete studies using only one group of participants, these included recreational adults (*n* = 11), elite juniors (*n* = 6), sub-elite adults (*n* = 5), elite adults (*n* = 3), and recreational juniors (*n* = 1). One study did not report athlete skill level. Of the six studies investigating officials, one compared elite and recreational adults, followed by recreational adults (n = 2), elite adults (*n* = 1), sub-elite adults (*n* = 1), and junior elite (*n* = 1). The most frequent sport investigated was soccer (*n* = 18), followed by handball (*n* = 10), and basketball (*n* = 5). Fourteen sports were investigated in three or fewer studies.

**Table 1:** Characteristics of included studies

Study	Technology type (Visual likeness)	Athletes or officials	Sport	Perceptual-cognitive skill	Study focus	Participant number	Sex	Skill level	Conclusions
Bideau et al. [21]	CAVE (Animated)	Athletes	Handball	DM, A	Quantitative (Assessment)	1	NR	NR	VR offered enough realism to initiate natural movement gestures in a handball setting
Bideau et al. [22]	CAVE (Animated)	Athletes	Handball	A, VA	Quantitative (Assessment)	8	NR	Elite	Identical throws in VR caused the goalkeeper to produce identical gestures
Boyer et al. [23]	360°VR (Real world)	Officials	Football (soccer), rugby, handball	DM	Qualitative	12	Both	Recreational (adult)	The reflectivity of the referees was suitable in the 360°VR condition due to first-person viewpoint
Correia et al. [24]	HMD (Animated)	Athletes	Rugby	DM	Quantitative (Assessment)	46	NR	Elite; Sub-elite; Recreational (adult); Other: Non-rugby players	VR was able to demonstrate effect of rugby expertise on perceiving and acting upon relevant game affordances
Craig et al. [25]	HMD (Animated)	Athletes	Football (soccer)	A	Quantitative (Assessment)	21	Male	Elite; Other: Non-soccer players	Football players can predict ball trajectory using advanced cues such as sidespin in a VR environment
Craig et al. [26]	HMD (Animated)	Athletes	Football (soccer)	DM	Quantitative (Assessment)	21	Both	Recreational (adult)	Intercepting a VR ball depends on the prospective control of movement where perception informs decisions about when and how to act
Discombe et al. [27]	360°VR (Real world)	Athletes	Cricket	A	Quantitative (Assessment)	18	Both	Recreational (adult)	Batters were able to predict landing location of ball more accurately in 360°VR compared to traditional videos
Rojas-Ferrer et al. [28]	HMD (Animated)	Athletes	Football (soccer)	DM, VS	Quantitative (Assessment)	24	Both	Recreational (adult)	Kinetic controllers are better than button-based input devices for DM simulations, and high presence measured in virtual environment
Fortes et al. [29]	360°VR (Real world)	Athletes	Football (soccer)	DM, VS	Quantitative (Intervention)	26	NR	Junior (elite)	Some benefits from using 360°VR for decision-making and visual search behaviour
Gray [30]	CAVE (Animated)	Athletes	Baseball	DM	Quantitative (Intervention)	80	Male	Junior (recreational)	Positive, near and far transfer of training of VR for baseball batting
Gray and Cañal-Bruland [31]	HMD (Animated)	Athletes	Baseball	DM, A, M&K	Quantitative (Assessment)	20	Male	Sub-elite	Experienced baseball players use situational probabilities and visual trajectory information in a flexible manner in VR
Gulec et al. [32]	HMD (Animated)	Officials	Football (soccer)	DM	Quantitative (Assessment)	5	NR	Recreational (adult)	VR can be used as a training tool to increase experience levels of football officials
Gürbüz and Murat [33]	HMD (Animated)	Athletes	Football (soccer)	DM	Quantitative (Intervention)	24	Male	Junior (elite)	VR can be used as an alternative training method for improving heading skills in football
Harris et al. [34]	HMD (Animated)	Athletes	Golf	VS	Quantitative (Intervention), Quantitative (Assessment)	58	Both	Elite; Recreational (adult)	The underlying skill structures learned in VR can be transferred to real-world, but the deficiencies of VR’s likeness will limit the use of VR
Harris et al. [35]	HMD (Animated)	Athletes	Golf	VS	Quantitative (Assessment)	127	Both	Recreational (adult)	It is possible to manipulate gaze without affecting performance in VR
Harris et al. [36]	HMD (Animated)	Athletes	Golf	VS	Quantitative (Intervention)	24	Both	Recreational (adult)	Training in VR led to improvements in gaze and motor behaviour following a short, VR intervention
Heilmann et al. [37]	CAVE (Animated)	Athletes	Football (soccer)	A, VA, M&K	Quantitative (Intervention)	82	Male	Junior (elite)	There were significant differences between age groups for cognitive abilities in the VR test, however, the changes following the intervention were unclear
Helm et al. [38]	HMD (Animated)	Athletes	Handball	A	Quantitative (Assessment)	27	Both	Recreational (adult)	Participants rely more on non-kinematic information such as contextual information, rather than kinematic cues
Höner et al. [39]	360°VR (Real world)	Athletes	Football (soccer)	DM	Quantitative (Assessment)	48	Male	Junior (elite)	360°VR can be used as a diagnostic tool for youth football player decision-making
Hosp et al. [40]	360°VR (Real world)	Athletes	Football (soccer)	VS	Quantitative (Assessment)	35	NR	Elite; Sub-elite; Recreational (adult); Junior (elite)	There were differences between skill levels in gaze behaviour within a 360°VR goalkeeping simulation
Kelly et al. [41]	HMD (Animated)	Athletes	Cricket	DM, A	Quantitative (Assessment)	28	Male	Elite; Recreational (adult)	VR environments with appropriate design can be effective tools for understanding expertise of decisions and actions
Kesilmis [42]	HMD (Animated)	Athletes	Handball	VA	Quantitative (Intervention)	70	Female	Other: Junior novice	Individual VR program applied in addition to handball training effectively improves the balance ability and reaction time
Kincaid et al. [43]	HMD (Animated)	Athletes	Baseball	DM	Mixed methods	25	Male	Sub-elite	VR does not improve DM, but participants report it to be a fun and useful training tool
Kittel et al. [44]	360°VR (Real world)	Officials	Australian football	DM	Quantitative (Assessment)	28	NR	Elite; Recreational (adult)	360 VR is a valid and reliable assessment tool of decision-making, and has higher game likeness ratings than match broadcast video
Kittel et al. [45]	360°VR (Real world)	Officials	Australian football	DM	Quantitative (Intervention)	32	NR	Recreational (adult)	360°VR was not better than traditional video, but had higher ratings of enjoyment/relevance, with no differences for concentration/effort
Kittel et al. [46]	360°VR (Real world)	Officials	Australian football	DM	Quantitative (Assessment)	21	NR	Elite	360°VR does not distinguish elite performance as ranked by on-field decision-making performance
Klatt et al. [47]	CAVE (Animated)	Athletes	Football (soccer)	DM, VS, M&K	Quantitative (Assessment)	27	Both	Recreational (adult)	In a CAVE environment, participants better view teammates and their positioning in the left field-of-view than the right
Klatt and Smeeton [48]	CAVE (Animated)	Athletes	Football (soccer)	DM	Quantitative (Assessment)	30	Both	Recreational (adult)	High physical load impacted DM accuracy in a CAVE environment more than moderate physical load
Le Noury et al. [49]	HMD (Animated)	Athletes	Tennis	DM, A	Quantitative (Assessment)	28	Both	Junior (elite)	Tennis VR provides a high level of presence with minimal negative effects
Limballe et al. [50]	HMD (Animated)	Athletes	Boxing, Mixed Martial Arts	A, VS	Quantitative (Assessment)	18	Both	Elite; Sub-elite; Recreational (adult)	Gaze-contingent blur influences interception performance and eye gaze behaviour
Loiseau-Taupin et al. [51]	360°VR (Real world)	Athletes	Boxing	VS	Quantitative (Assessment)	32	Both	Recreational (adult)	360VR leads to shorter fixation durations and greater head excursions in boxing and had greater immersion and presence
Luke et al. [52]	360°VR (Real world)	Athletes	Surfing	VS	Quantitative (Assessment)	24	Both	Elite; Recreational (adult)	360°VR can be used to examine visual search behaviour for surfing
Magnaguagno et al. [53]	CAVE (Real world)	Athletes	Handball	DM, A, M&K	Quantitative (Intervention)	24	Male	Elite; Sub-elite	Expert players were able to make more accurate decisions and use cues better than sub-elite players
Magnaguagno et al. [54]	CAVE (Real world)	Athletes	Handball	DM, A, M&K	Quantitative (Intervention)	57	Male	Junior (elite); Junior (recreational)	Providing individuals with uncertain information in a CAVE DM task does not enhance nor harm performance
Magnaguagno et al. [55]	CAVE (Real world)	Athletes	Handball	DM, A, M&K	Quantitative (Intervention)	81	Male	Junior (elite)	This study furthered the data analysis of two previous studies by the same author, identifying that experience is a key factor for decision-making accuracy in the VR environment than explicitly generated knowledge
Mann et al. [56]	HMD (Animated)	Athletes	Tennis	VS	Quantitative (Assessment)	23	NR	Recreational (adult)	A VR tennis simulation can identify where participants are predicting the ball to bounce
Manzanares et al. [57]	CAVE (Animated)	Athletes	Sailing	VS	Quantitative (Assessment)	20	Both	Junior (elite); Junior (recreational)	The VR sailing simulation was able to differentiate between high and low-ranked sailors in their use of visual information
Pagé et al. [58]	360°VR (Real world)	Athletes	Basketball	DM	Quantitative (Intervention)	27	Both	Sub-elite	360°VR training developed DM, particularly for untrained plays
Panchuk et al. [59]	360°VR (Real world)	Athletes	Basketball	DM	Quantitative (Intervention)	18	Both	Junior (elite)	360°VR training developed DM on-court performance, for trained males
Petri et al. [60]	HMD (Animated)	Athletes	Karate	DM, VA	Quantitative (Intervention)	15	Both	Sub-elite; Recreational (adult); Junior (elite); Junior (recreational)	VR training led to more movement in karate training, and participants were highly motivated
Polikanova et al. [61]	HMD (Animated)	Athletes	Hockey	A, VA	Quantitative (Assessment)	20	NR	Elite; Other: Non-hockey players (wrestlers)	VR can lead to differences between elite hockey and non-hockey players in stance and response time, but not skill performance
Richard et al. [62]	360°VR (Real world)	Athletes	Basketball	DM	Quantitative (Assessment)	11	Both	Sub-elite	360°VR videos were perceived as faster compared to computer screen videos, and perceived to be more like real-world performance
Ritter et al. [63]	HMD (Animated)	Athletes	Karate	DM, VA	Quantitative (Assessment)	27	Both	Elite; Junior (elite)	Response time and decisions made are similar for karate athletes in both VR and the real world
Romeas et al. [64]	CAVE (Animated)	Athletes	Badminton	DM, A, M&K	Quantitative (Intervention)	55	Both	Sub-elite; Other: nonathlete adults	It is important to combine perceptual-cognitive and motor elements into a VR simulation for a more representative training tool
Romeas et al. [65]	360°VR (Real world)	Athletes	Boxing	DM, VA	Quantitative (Intervention)	6	Both	Elite	There was good acceptance of VR by athletes as an individual training option
Shimi et al. [66]	HMD (Animated)	Athletes	Football (soccer)	DM, A, VA	Quantitative (Assessment)	100	Both	Recreational (adult); Other: University students—not current players	VR can induce natural individual differences in perceptual-cognitive skills
Tanaka et al. [67]	MR (Animated)	Athletes	Karate	VA	Quantitative (Assessment)	11	Male	Elite; Other: Recreational (adult)	Mixed reality for karate can reduce reaction time but does not appear to enhance learning
Tsai et al. [68]	HMD (Animated); 360°VR (Real world)	Athletes	Basketball	VA	Quantitative (Intervention)	45	Both	Recreational (adult)	360°VR and VR both have similar knowledge acquisition, presence, as response time
Valkanidis et al. [69]	HMD (Animated)	Athletes	Football (soccer)	DM, A	Quantitative (Assessment)	25	NR	Sub-elite; Recreational (adult); Other: No prior experience goalkeeping	Visual occlusion of the initial ball trajectory by a ‘wall’ of players’ negatively affects performance in a football goalkeeping simulation
van Biemen et al. [70]	HMD (Animated)	Officials	Football (soccer)	DM, VS	Quantitative (Assessment)	15	Male	Sub-elite	Visual search and DM in VR are similar to real-world, particularly compared to screen-based approaches
van Leeuwen et al. [71]	CAVE(Animated)	Athletes	Racing	VS, VA	Quantitative (Assessment)	17	Male	Elite; Other: Non-racing drivers	Race car drivers performed better in the simulation than non-race drivers, and there were differences in visual search behaviours
Vignais et al. [72]	CAVE (Animated)	Athletes	Handball	DM, A	Quantitative (Assessment)	10	Male	Elite	In a VR environment, athletes track body cues to predict where the ball is traveling
Vu et al. [73]	HMD (Animated)	Athletes	Football (soccer)	VS	Quantitative (Assessment)	33	Both	Elite; Sub-elite; Other: Non-soccer players	VR can distinguish between high level and non-football players’ ability to track multiple players
Watson et al. [74]	HMD (Animated)	Athletes	Rugby	DM	Quantitative (Assessment)	14	Both	Other: Non-rugby playing adults	Participants were able to use information in the VR environment to predict whether to pass
Wirth et al. [75]	HMD (Animated)	Athletes	Football (soccer)	M&K	Quantitative (Assessment)	28	Both	Sub-elite	VR can be a valid tool to determine characteristics between skill levels
Wood et al. [76]	HMD (Animated)	Athletes	Football (soccer)	VA	Quantitative (Assessment)	51	Both	Elite; Junior (elite); Recreational (adult)	VR demonstrates construct validity between different skill levels
Wu et al. [77]	HMD (Animated)	Athletes	Skiing	VS	Quantitative (Assessment)	7	Both	Elite; Junior (elite)	Environmental factors in a VR environment such as photographers, staff can increase head and eye movement

Perceptual cognitive skills are categorised by Hodges et al. [18]

HMD, head mounted display; VR, virtual reality; MR, mixed reality; DM, decision-making; A, anticipation; VA, visual attention; VS, visual search; M&K, memory & knowledge; NR, not reported

### XR Technology Types and Applications

An array of XR technologies was utilised in the included studies. Most of the included research utilised HMD (Animated) (*n* = 29), followed by 360°VR (Real World) (*n* = 15), CAVE (Animated) (*n* = 9), and CAVE (Real World) (*n* = 3). One study explored mixed reality [67], and one study compared HMD (Animated) and 360°VR (Real world) [68]. Therefore, 40 studies used Animated/Virtual environments, whereas 17 studies used Real world video in an XR environment. The applications within each technology type are detailed in Table 2. Across all technology types decision-making was the most prevalent skill developed (across *n* = 34 studies), followed by visual search (*n* = 16), anticipation (*n* = 15), visual attention (*n* = 12), memory & knowledge (*n* = 8).

**Table 2:** Applications of XR technologies

Characteristic	XR technology type				Total
	HMD (Animated) (*n* = 29)	360°VR (Real world) (*n* = 15)	CAVE (Animated) (*n* = 9)	CAVE (Real world) (*n* = 3)	
Perceptual cognitive skill practiced					
Decision-making	26	10	6	3	45
Visual search	9	4	3	/	16
Anticipation	9	1	5	/	15
Visual attention	7	2	3	/	12
Memory & knowledge	3	/	4	1	8
Sport					
Soccer	11	4	3	/	18
Handball	2	1	4	3	10
Basketball	1	4	/	/	5
Baseball	2	/	1	/	3
Australian football	/	3	/	/	3
Rugby	2	1	/	/	3
Karate	2	/	/	/	2
Boxing	1	2	/	/	3
Golf	3	/	/	/	3
Tennis	2	/	/	/	2
Cricket	1	1	/	/	2
Skiing	1	/	/	/	1
Sailing	/	/	1	/	1
Surfing	/	1	/	/	1
Badminton	/	/	1	/	1
Athletes or officials					
Athletes	29	12	10	3	51
Officials	2	4	/	/	6

### Study Design Characteristics

Of the 57 studies included in the systematic review, 38 were Quantitative (Assessment) studies, 16 were Quantitative (Intervention) only, one utilised Qualitative method, one study used mixed methods (Quantitative Intervention and Qualitative) and 1 study was a combined Quantitative (Intervention and Assessment) design.

As seen in Table 3, both control/placebo and intervention comparison groups were used most commonly for intervention-only studies (*n* = 10). The duration of intervention studies ranged from single sessions to 8 weeks. Most intervention studies were 4–6 weeks in duration (*n* = 6), with single session and 3–4 sessions per week being most frequent.

**Table 3:** Study design characteristics of intervention only studies (*n* = 16)

Characteristics	No. of studies
Comparison group	
Control/placebo only	3
Intervention only	7
Control/placebo and intervention	6
Session frequency (per week)	
Single session	5
1–2	4
3–4	5
NR	2
Intervention Duration	
Single session	5
1–3 weeks	1
4–6 weeks	6
> 6 weeks	2
NR	2

NR, not reported

## Discussion

This scoping review has sought to provide a comprehensive investigation of how XR technology has been used to examine and enhance perceptual-cognitive skills within sport and has summarised key study characteristics. This builds on the work of previous reviews by quantifying aspects of the literature such as participant and population demographics, technology used, perceptual-cognitive skills examined, and intervention characteristics. Fifty-seven studies were included in the final review. Whilst there were some common approaches taken (e.g., HMD with animated scenarios), as well as specific sports (e.g. soccer) and samples (participants of both sexes), the review highlights a diverse breadth of the literature. Included studies were found to investigate perceptual-cognitive skills using HMDs, CAVE systems, and 360°VR, and examined a wide range of skill levels (including that of officials) across 15 different sports, with soccer and handball the most explored.

Findings from the present review indicate that XR technologies have been most frequently adopted in soccer, which is logical given the global appeal of the sport and the considerable resources that many soccer organisations have available to invest in XR technology [78]. This may also reflect the high level of desire or motivation of key stakeholders in the sport of soccer to explore new ways of enhancing decision making skill. It is perhaps surprising, though, that more studies have not focused on baseball (*n* = 3) or cricket (*n* = 2) considering the prevalence of research related to perceptual-cognitive skills in these sports [18]. A key finding of the present review was that included studies tended to apply XR to topics of *existing* interest (e.g., decision-making in soccer), rather than utilising the advantages of XR to identify *new* topics of interest. For instance, XR would appear to provide a prime opportunity to study the gaze behaviour of baseball/cricket batters under specific environmental conditions (e.g. facing unique pitches/deliveries) that are difficult to replicate precisely in the real world.

Another key finding of this review was that most studies focused on athletes (*n* = 51 studies), with significantly less focusing on officials (*n* = 6 studies). However, 360°VR may offer an opportunity for more research to be done with officials [79], with more studies exploring 360°VR than virtual environments in this population. Indeed, an important aspect of officials' expertise is their ability to identify and read key cues in the environment or process information effectively to make accurate decisions [80]. This is particularly important in sports like cricket where the umpire is stationary while processing the information that informs their decisions [81]. Given that 360°VR has the capacity to represent highly representative visual and audio information, this represents a viable area for future officiating research to be conducted in utilising this XR modality [79]. Notably, sports that include interactor officials with high movement and perceptual-cognitive demands [82], such as soccer referees and Australian football umpires, require the officials to constantly adapt their positioning and movement in order to gather information required to make an accurate decision. However, 360°VR is not capable of replicating this type of perception–action coupling, and therefore caution should be taken if utilising 360°VR for training decision-making of officials in dynamic environments. This may explain why 360°VR was not necessarily an effective tool for decision-making development in Australian football umpires [45], but can be used as a reflective tool for this skill for soccer and rugby referees [23]. Thus, further research using XR in officiating populations could use dynamic 360°VR environments, and/or build on the seminal work of van Biemen et al. [70] exploring VR for soccer referees.

Quantitative (Assessment) study designs of XR technologies (e.g. validity/reliability, identifying gaze behaviour characteristics) was found to be an area of priority in the studies included in the current review (*n* = 38) [79, 82]. This is to be expected given that XR research within sport is still in its infancy. Indeed, 67% of studies in this review were published in 2020 or later, whilst only three studies were pre-2010, highlighting the rate of growth in this research area. It is interesting to note, however, that there have been recent calls for further research to test the suitability of XR technology for testing and training decision-making [8]. Some Quantitative (Assessment) studies included in this review were able to identify expertise effects [41, 53, 57], determine visual search characteristics [52, 56, 70], and provide evidence of similar skill performance in both the XR environment and real-world performance [24, 34, 63]. This is particularly important given that a lack of empirical evidence has been suggested to be a key barrier for adoption of the technology [3, 83].

Within this review, 16 were intervention studies. These studies used a variety of mechanisms to assess the changes in performance, such as in-competition performance [30, 36], small-sided games [29, 58, 59], or assessment used the same XR technology [45]. This highlights that more work needs to be done to assess evidence of far transfer, where training using the XR technology leads to better in-competition performance [84, 85]. There are promising results for the use of this technology but given the variety of mechanisms to assess the effectiveness of these interventions, it is unclear without a meta-analysis how effective this technology is. This would build on the work of the current review and that of Yunchao et al. [9], who addressed only decision-making training. Nevertheless, there is clearly a need for more intervention studies which examine the effectiveness of XR to enhance perceptual-cognitive skills in sport. Such work is crucial as it is the training of these skills which coaches consider to be the preferred use case for XR [86], and greater evidence would help determine the provision of resources towards the technology within sports organisations [78].

A promising finding from this review is that many studies reported XR to be similar to competition, which is an important concept of these simulations demonstrating strong ecological validity and representativeness [15, 87]. For example, a tennis VR simulation led to heightened levels of presence [44], a 360°VR Australian football officiating simulation was rated as more game-like than video [39], and similarly for 360°VR in basketballers [57]. VR was also reported to be a fun and engaging tool for athlete development in several studies [43, 45, 60]. Interestingly, one study provided a comparison between HMD (Animated) and 360°VR in basketball players [63], and found both have similar levels of presence, but more work is needed to be done as this was the only study identified to provide a comparison between multiple XR technologies. How comparable XR is to real-world competition requires further investigation, with some studies reporting that the limitations of VR’s likeness will limit its use [34]. Overall, the findings from this review point to XR increasingly becoming a representative training and assessment tool for perceptual-cognitive skills, which is a key component for its effectiveness [2, 14].

Ultimately, the findings of this scoping review can have important implications for stakeholders within sport who are considering implementing XR technologies with their athletes or officials. By providing an overview of the current literature, this review should be able to guide stakeholders to studies which are specific and relevant to their needs, enabling them to become more informed about XR technology in sport. Furthermore, by collating the approaches taken within the literature, stakeholder confidence can be built in certain methods of implementation; for instance, in the use of XR to examine decision-making within soccer, or the use of 360°VR to train the perceptual-cognitive skills of officials.

Researchers in the field should also benefit from this review by identifying gaps within the literature. As highlighted previously, there has been limited research done examining the use of XR in sports such as baseball and cricket. This omission goes beyond the valid technological limitations that currently exist regarding accurate simulation of bat-ball contact within such sports, as highlighted by a recent paper in which six use cases were outlined for the implementation of VR within baseball [88]. Similarly, longitudinal research in the area is currently lacking, and whilst such criticism can be levelled at the majority of training modalities within sport, it is particularly important to apply to XR, given its status as a new, technological innovation that may simply be the ‘flavour of the month’ [89]. Thus, based on the findings of our review, we would encourage researchers to examine the impact of a season-long XR intervention on performance (in the form of both perceptual-cognitive skills and real-world competition metrics) in interceptive sports such as baseball, cricket, and tennis.

There are several areas for further research in this area to inform practice. Twenty-six percent of studies within this review had male-only participants, 51% had both males and females, 21% did not report sex, and 2% were female only. There is some evidence that females experience cybersickness within virtual reality to a greater extent than males, which could conceivably impact the findings of XR research with such populations [90], and therefore the dominance of male participants within this review may not be presenting the full picture for XR findings. In comparison to research assessing the validity and reliability of XR technology, there was limited research from both a qualitative perspective and intervention design in comparison. More qualitative research in this area would provide a deeper understanding into *how* these technologies can be used to develop perceptual-cognitive skills. In addition, more interventions are required and over a longer period, to determine the effectiveness of these technologies for developing perceptual-cognitive performance in sport. The studies included in this review were relatively low in sample size, with some included studies indicating this as a limitation of their research [45, 53, 59]. Recent commentary has suggested that this is a common trend in the sport science literature [91, 92], and low sample sizes may influence the replicability of the research. Therefore, future research should increase sample sizes in this area, whilst balancing the pragmatic challenges of collecting data in an applied sport setting, and the financial cost of XR equipment along with its limitations of accessibility [59].

It is important to acknowledge that our work is not without its limitations. The purpose of our scoping review was to map the existing perceptual-cognitive XR literature for stakeholders within sport so that trends within the literature could be identified and common methodological approaches could be outlined. Thus, we have not attempted to synthesise the findings of the included studies or provide an assessment of the quality of the research. Future research could explore subtopics within this review (e.g. XR interventions, or XR within officiating populations) and provide a deeper overview of the study quality (i.e., methods, reporting) in a subset of the literature.

## Conclusions

To conclude, our scoping review of XR technology to examine perceptual-cognitive skills in sport included 57 studies, most of which were published in the last 4 years. This emerging research topic has produced a diverse branch of work, though certain methodological approaches appear more common than others; most notably, Quantitative (Assessment) study designs and few intervention studies, research focusing on decision-making as the predominant perceptual-cognitive skill investigated, studies using HMDs, and studies with football (soccer) athletes. In addition, while there is growing evidence of this technology in open team-based sports such as football and handball, more work could be undertaken with other populations, such as athletes from interceptive sports (cricket, baseball) and officiating groups. Nonetheless, this technology appears to be an engaging and representative technology for developing athletes and officials, given the findings of this review. Future work should address the less-researched aspects identified from this review, such as the lack of intervention designs and further incorporation of theoretical frameworks to support the design of the studies. These knowledge gaps hold important implications for practitioners, in that further work is required to understand the effectiveness of these technologies for developing perceptual-cognitive skill through an intervention. To summarise, this review provides a succinct overview of the implementation of XR technology within the domain of perceptual-cognitive skills by technology type, sport, skill(s) investigated, study design, for both practitioners and academics to further understand this growing field of research.

## Data Availability

All data generated or analysed during this study are included in this published article.

## Abbreviations

XR: Extended reality
VR: Virtual reality
MR: Mixed reality
RLD: Representative learning design
ALP: Affective learning design
MPTF: Modified perceptual training framework
HMD: Head mounted display
DM: Decision-making
A: Anticipation
VA: Visual attention
VS: Visual search
M&K: Memory & knowledge
NR: Not reported
